# Effects of colchicine adjuvant therapy on disease control, serum NALP3, sICAM-1, MMP-9 and MMP-13 in patients with coronary heart disease and acute gout attack

**DOI:** 10.5937/jomb0-51326

**Published:** 2025-01-24

**Authors:** Wang Bingxun, Liu Yongqing, Han Wenya, Li Bing, Jie Pang, Yang Wenwen, Ma Zengcai, Xu Zesheng

**Affiliations:** 1 Cangzhou Central Hospital, Department of Cardiology, Cangzhou, China; 2 Hebei Medical University, Cangzhou Central Hospital, Shijiazhuang, China; 3 Cangzhou Central Hospital, Department of Rheumatology, Cangzhou, China

**Keywords:** colchicine, coronary heart disease, acute gout attack, nucleotide binding oligomerization domain-like receptor protein 3, soluble intercellular adhesion molecule1, matrix metalloproteinase-9, matrix metalloproteinase13, cardiac function, kolhicin, koronarna bolest srca, akutni napad gihta, receptorski protein 3 nalik domeni oligomerizacije vezivanja nukleotida, rastvorljivi međućelijski adhezioni molekul-1, matriks metaloproteinaza-9, matriks metaloproteinaza-13, srčana funkcija

## Abstract

**Background:**

To investigate the impact of colchicine adjuvant therapy on disease control and serum levels of nucleotide-binding oligomerization domain-like receptor (NALP) 3, soluble intercellular adhesion molecule (sICAM)-1, matrix metalloproteinase (MMP)-9, and MMP13 in patients with coronary heart disease (CHD) complicated by acute gout attacks.

**Methods:**

Ninety-two patients with CHD and acute gout attacks admitted to our hospital from October 2021 to January 2023 were randomly divided into an observation group and a control group, with 46 patients in each group. The control group received conventional treatment, while the observation group received colchicine adjuvant therapy on top of the control group's treatment for 7 days. Clinical efficacy in both groups was assessed. Before and after treatment, cardiac function indicators (left ventricular ejection fraction (LVEF), left ventricular end-diastolic diameter (LVEDD), left ventricular posterior wall thickness (LVPWT)), vascular endothelial function indicators (sICAM-1, endothelin-1 (ET-1), and vascular endothelial growth factor (VEGF)), inflammatory factors (NALP3, MMP-9, MMP-13) levels, changes in immune cell populations (CD3+ lymphocytes, CD3+CD4+ lymphocytes, CD3+CD8+ lymphocytes ratio, and CD3+CD4+/CD3+CD8+ ratio) were compared, and the incidence of adverse reactions was recorded. Three months after treatment, the occurrence of major adverse cardiovascular events was also recorded.

**Results:**

The total effective rate in the observation group was significantly higher than that in the control group (93.48% vs 79.07%) (P<0.05). After treatment, the levels of NALP3, MMP-9, and MMP-13 in both groups decreased, with the observation group being lower than the control group (P<0.05). After treatment, LVPWT and LVEDD levels in the observation group were lower than those in the control group, and LVEF was higher (P<0.05). After treatment, the levels of ET-1 and sICAM-1 in the observation group were lower than those in the control group, and VEGF levels were higher (P<0.05). After treatment, the proportions of CD3+ lymphocytes, CD3+CD4+ lymphocytes, and CD3+CD4+/CD3+CD8+ ratio were significantly higher in the observation group than in the control group (P<0.05). There was no significant difference in the incidence of adverse reactions between the two groups (P>0.05). The occurrence of major adverse cardiovascular events in the observation group was lower than that in the control group (2.17% vs 13.04%).

**Conclusions:**

Colchicine adjuvant therapy improves the efficacy of CHD patients with acute gout attacks, helps improve cardiac function and vascular endothelial function, reduces serum levels of NALP3, sICAM-1, MMP-9, and MMP-13, enhances patient immunity, and controls disease progression.

## Abstract (sr)

**Uvod:**

Cilj je bio da se istraži uticaj adjuvantne terapije kolhicinom na kontrolu bolesti i nivoe u serumu receptora sličnog domenu oligomerizacije koji se vezuje za nukleotide (NALP) 3, rastvorljivog intercelularnog adhezionog molekula (sICAM)-1, matriks metaloproteinaze (MMP)-9 i MMP-13 kod pacijenata sa koronarnom bolešču srca (CHD) komplikovanom akutnim napadima gihta.

**Metode:**

Devedeset i dva pacijenta sa KBS i akutnim napadima gihta primljena u našu bolnicu od oktobra 2021. do januara 2023. nasumično su podeljena u posmatračku i kontrolnu grupu, sa po 46 pacijenata u svakoj grupi. Kontrolna grupa je primila konvencionalni tretman, dok je posmatračka grupa primala pomočnu terapiju kolhicinom pored tretmana kontrolne grupe tokom 7 dana. Procenjena je klinička efikasnost u obe grupe. Pre i posle tretmana, indikatori srčane funkcije (ejekciona frakcija leve komore (LVEF), end-dijastolni prečnik leve komore (LVEDD), debljina zadnjeg zida leve komore (LVPVT)), indikatori funkcije vaskularnog endotela (sICAM-1, endotelin-1 ( ET-1) i faktor rasta vaskularnog endotela (VEGF)), nivoi inflamatornih faktora (NALP3, MMP-9, MMP-13), promene u populaciji imunih čelija (CD3+ limfociti, CD3 + CD4+ limfociti, odnos CD3+CD8+ limfocita, i CD3 + CD4+/CD3+CD8+ odnos) i zabeležena je učestalost neželjenih reakcija. Tri meseca nakon tretmana zabeležena je i pojava velikih neželjenih kardiovaskularnih događaja.

**Rezultati:**

Ukupna efektivna stopa u posmatranoj grupi bila je značajno viša od one u kontrolnoj grupi (93,48% naspram 79,07%) (P<0,05). Nakon tretmana, nivoi NALP3, MMP-9 i MMP-13 u obe grupe su se smanjili, pri čemu je posmatrana grupa bila niža od kontrolne grupe (P<0,05). Nakon tretmana, nivoi LVPVT i LVEDD u posmatranoj grupi bili su niži od onih u kontrolnoj grupi, a LVEF viši (P<0,05). Nakon tretmana, nivoi ET-1 i sICAM-1 u posmatranoj grupi bili su niži od onih u kontrolnoj grupi, a nivoi VEGF-a viši (P<0,05). Nakon tretmana, proporcije CD3+ limfocita, CD3 + CD4+ limfocita i odnos CD3 + CD4+/CD3 + CD8+ bili su značajno veči u posmatranoj grupi nego u kontrolnoj grupi (P<0,05). Nije bilo značajne razlike u incidenci neželjenih reakcija između dve grupe (P>0,05). Pojava velikih neželjenih kardiovaskularnih događaja u posmatranoj grupi bila je manja nego u kontrolnoj grupi (2,17% prema 13,04%).

**Zaključak:**

Adjuvantna terapija kolhicinom poboljšava efikasnost pacijenata sa KBS sa akutnim napadima gihta, pomaže u poboljšanju funkcije srca i vaskularnog endotela, smanjuje nivoe NALP3, sICAM-1, MMP-9 i MMP-13 u serumu, poboljšava imunitet pacijenata i kontroliše progresiju bolesti.

## Introduction

Coronary heart disease (CHD), as a cardiovascular disease, is the most common type of organ damage caused by atherosclerosis, predominantly affecting the middle-aged and elderly population. It has a high incidence, disability rate, and mortality rate [1]. Given the close association between the occurrence of cardiovascular diseases and high uric acid levels, and because high uric acid is a direct cause of acute gout attacks [2], CHD patients often experience acute gout attacks, significantly worsening their condition. Therefore, early control and active treatment have a positive impact on improving the patient’s condition and promoting recovery.

Currently, the clinical treatment of CHD patients with acute gout attacks mainly focuses on antiplatelet therapy, lipid regulation, control of risk factors, and uric acid reduction. However, in recent years, in creasing evidence suggests that inflammation plays a significant role in the formation and progression of atherosclerosis [3]. It is thus speculated that anti-inflammatory treatment will become a new direction in the treatment of CHD. Previous research by Ridker PM and others has also shown that anti-inflammatory treatment can improve the prognosis of CHD patients [4].

Colchicine is a lipophilic alkaloid extracted from autumn crocus and has been approved for use in the treatment of acute gout and familial Mediterranean fever, showing significant efficacy [5]. As an anti-inflammatory drug, it has been found to have broad prospects in the secondary prevention of CHD, promoting plaque stability, reducing acute plaque events, and major adverse cardiovascular events [6] [7].

Given these considerations, this study will use colchicine as adjuvant therapy for CHD patients with acute gout attacks to investigate its effects on disease control, serum levels of nucleotide-binding oligomerization domain-like receptor protein (NALP) 3, soluble intercellular adhesion molecules (sICAM)-1, matrix metalloproteinase (MMP)-9, MMP-13, and immune function cells.

## Materials and methods

This study was approved by the ethics committee of Cangzhou Central Hospital (approval number: 2022-051-02(z)). Signed written informed consents were obtained from the patients and/or guardians.

### Basic characteristics

A total of 92 patients with CHD complicated by acute gout attacks, who were admitted to our hospital from October 2021 to January 2023, were selected for this study. They were divided into an observation group and a control group, with 46 patients in each group. Control group: Male/Female = 39/7, age (average age) 53 to 79 (66.28 ± 4.43) years, New York Heart Association (NYHA) classification: I/II/III/IV = 6/15/18/7 cases, body mass index (BMI, average BMI) 21 to 26 (23.24 ± 1.12) kg/m^2^; Observation group: Male/Female = 40/6, age (average age) 53 to 79 (66.59 ± 4.27) years, NYHA classification: I/II/III/IV = 4/13/17/12 cases, BMI (average BMI) 21 to 26 (23.51 ± 1.05) kg/m^2^. There were no significant differences in baseline data between the two groups (P > 0.05). This study was approved by the hospital ethics committee. Inclusion criteria: All patients met the diagnostic criteria for CHD [8] and acute gout attack [9]. Patients and their families were informed about this study. Exclusion criteria: Patients with allergies to the medications used in this study; patients with psychiatric disorders; patients with other types of cardiovascular diseases; patients with significant liver or kidney dysfunction; patients with autoimmune diseases.

### Drug treatment

The control group received standard treatment, which included: Aspirin (Italy Bayer HealthCare Manufacturing S.r.l., National Drug Approval HJ20160685, 100 mg×30 tablets): 1 tablet per dose, once daily, taken orally after meals. Clopidogrel bisulfate tablets (Lepu Pharmaceutical Co., Ltd., National Drug Approval H20123116, 75 mg×10 tablets): 1 tablet per dose, once daily. Atorvastatin calcium tablets (Fujian Dongrui Pharmaceutical Co., Ltd., National Drug Approval H20193043, 10 mg×28 tablets): 1 tablet per dose, once daily. Telmisartan tablets (Beijing Fuyuan Pharmaceutical Co., Ltd., National Drug Approval H20050996, 40 mg×14 tablets): 1 tablet per dose, once daily. Bisoprolol fumarate tablets (Beijing Huasu Pharma ceutical Co., Ltd., National Drug Approval H10970082, 5 mg×10 tablets): 1 tablet per dose, once daily. Isosorbide dinitrate tablets (Lunan Better Pharmaceutical Co., Ltd., National Drug Approval H10940039, 20 mg×48 tablets): 2–3 times daily, 10–20 mg per dose. The observation group, in addition to the medications administered to the control group, received adjunctive therapy with colchicine (Guangdong Bidi Pharmaceutical Co., Ltd., National Drug Approval H20113208, 0.5 mg×20 tablets). The dosage was 0.5mg per dose, three times daily initially, and was adjusted to 0.5 mg per dose, twice daily once the condition improved. Both groups continued their medication regimen for 7 days.

### Observation parameters

Clinical Efficacy: At the end of the treatment, the clinical efficacy was evaluated based on reference to previous literature [10] and categorized into three groups: significant efficacy, efficacy, and inefficacy. Significant efficacy: Patients exhibited a significant improvement in clinical symptoms, good recovery of joint function, nearly normal electrocardiogram (ECG) results, and a return to nearly normal laboratory indicator levels. Efficacy: Patients experienced improvements in clinical symptoms and joint function, with ECG and laboratory indicators showing improvement. Inefficacy: Patients did not show improvements in clinical symptoms, joint function, ECG results, or laboratory indicator levels, and may have even worsened. The total effective rate was calculated as the sum of the significant efficacy rate and the efficacy rate.

Inflammatory Factors: Prior to treatment and at the end of treatment, fasting venous blood samples (3 mL) were collected from the patients. After centrifugation (3000 r/min, 10 min, 10 cm radius), the upper clear liquid was collected and stored at -40°C for further analysis. Enzyme-linked immunosorbent assay (ELISA) was used to measure the levels of NALP3, MMP-9, and MMP-13. The reagents and kits were purchased from Shanghai XunYa Biotechnology Co., Ltd., and the measurements were performed strictly following the manufacturer’s instructions.

Cardiac Function: Before treatment and at the end of treatment, a color Doppler ultrasound machine (Instrument: GE, LOC-ZQ9, USA) was used to measure the left ventricular posterior wall thickness (LVPWT), left ventricular end-diastolic diameter (LVEDD), and left ventricular ejection fraction (LVEF) of the patients.

Endothelial Function: Before treatment and at the end of treatment, fasting venous blood samples (3 mL) were collected from the patients. After centrifugation (3000 r/min, 10 min, 10 cm radius), the upper clear liquid was collected and stored at -40°C for further analysis. Enzyme-linked immunosorbent assay (ELISA) was used to measure the levels of endothelin-1 (ET-1), soluble intercellular adhesion molecule-1 (sICAM-1), and vascular endothelial growth factor (VEGF). The reagents and kits were purchased from Shanghai Enzyme Research Bio technology Co., Ltd., and the measurements were performed strictly following the manufacturer’s instructions.

Detection of Immune Function Cells: Before and after treatment, 3 mL of peripheral blood was collected from patients and placed in EDTA anticoagulant tubes. From each blood sample, 100 μL was taken and 5 μL of CD45-Percp, CD3-FITC, CD8-PE, and CD4-APC monoclonal antibodies were added separately. After incubation in the dark for 30 minutes, 1 mL of lysing reagent was added to lyse red blood cells. After thorough mixing and centrifugation, the supernatant was removed. The cells were washed twice with PBS and then analyzed using the BD FACS Canto flow cytometer to detect changes in the proportions of immune function cells, including CD3+ lymphocytes, CD3+CD4+ lymphocytes, and CD3+CD8+ lymphocytes, in peripheral blood before and after treatment. The immune function index (CD3+CD4+/CD3+CD8+ ratio) was also calculated.

Adverse Reactions: Adverse reactions occurring during the treatment period in both groups were recorded. These primarily included nausea, vomiting, headache, gastrointestinal bleeding, digestive tract bleeding, diarrhea, etc. If a patient experienced multiple adverse reactions, only the most severe one was recorded.

Major Cardiovascular Events: Three months after the end of treatment, both groups were followed up to record the occurrence of major cardiovascular events, including cardiovascular death, myocardial infarction, and stroke.

### Statistical analysis

Data processing was performed using Statistic Package for Social Science (SPSS) 22.0 software (IBM, Armonk, NY, USA). For cardiac function, vascular endothelial function, and inflammatory factor levels, which all followed a normal distribution and are represented as (mean ± standard deviation), differences between groups were analyzed using the two-sample independent t-test. For comparisons within each group before and after treatment, paired t-tests were used. Categorical data such as adverse reactions, gender, and efficacy were presented as (n (%)), and chi-square tests were employed. A significance level of P < 0.05 was considered statistically significant.

## Results

### Comparison of total effective rates between the two groups

The total effective rate in the observation group was significantly higher than that in the control group (93.48% vs. 79.07%) (P < 0.05). Detail information was shown in Table 1.

**Table 1:** Comparison of Total Effective Rates between the Two Groups (n (%), n=46).

Group	Significant<br>efficacy	Efficacy	Inefficacy	total<br>effective rate
Observation<br>group	29	14	3	93.48
Control<br>group	21	16	9	79.07
χ^2^				3.955
P-value				0.047

### Comparison of inflammatory factor levels between the two groups

Before treatment, there were no significant differences in NALP3, MMP-9, and MMP-13 levels between the two groups (P > 0.05). After treatment, the levels of NALP3, MMP-9, and MMP-13 decreased in both groups, with the observation group showing lower levels compared to the control group (P < 0.05). Detail information was shown in Table 2.

**Table 2:** Comparison of Inflammatory Factor Levels between the Two Groups (x̄±s, n=46). *Compared to before treatment, P<0.05; #compared to the control group, P<0.05.

Group	NALP3 (ng/L)		MMP-9 (ng/mL)		MMP-13 (pg/mL)	
	Before treatment	After treatment	Before treatment	After treatment	Before treatment	After treatment
Observation<br>group	9.64±2.13	7.15±1.06^*#^	187.58±26.35	143.51±14.70^*#^	105.64±9.78	82.51±5.13^*#^
Control group	9.57±2.08	8.49±1.23^*^	188.62±27.81	159.08±16.77^*^	105.24±9.11	90.75±6.38^*^
*T*	0.159	5.597	0.184	4.732	0.203	6.827
P-value	0.874	<0.001	0.854	<0.001	0.840	<0.001

### Comparison of cardiac function between the two groups

Before treatment, there were no significant differences in LVPWT, LVEDD, and LVEF levels between the two groups (P > 0.05). After treatment, the LVPWT and LVEDD levels in the observation group were lower than those in the control group, while the LVEF was higher in the observation group compared to the control group (P < 0.05). Detail information was shown in Table 3.

**Table 3:** Comparison of Cardiac Function between the Two Groups (x̄±s, n=46). ^*^Compared to before treatment, P<0.05; ^#^compared to the control group, P<0.05.

Group	LVPWT (mm)		LVEDD (mm)		LVEF (%)	
	Before treatment	After treatment	Before treatment	After treatment	Before treatment	After treatment
Observation<br>group	15.14±2.28	8.37±1.14^*#^	53.54±3.61	44.30±2.15^*#^	40.65±2.33	53.80±2.34^*#^
Control group	15.29±2.07	10.92±1.23^*^	53.93±3.26	48.71±2.12^*^	40.71±2.48	47.22±1.09^*^
*T*	0.330	10.313	0.544	9.906	0.120	17.288
P-value	0.742	<0.001	0.588	<0.001	0.905	<0.001

### Comparison of endothelial function between the two groups

After treatment, the levels of ET-1 and sICAM-1 decreased, while the level of VEGF increased in both groups. Additionally, the observation group had lower levels of ET-1 and sICAM-1 compared to the control group, and higher levels of VEGF (P < 0.05). Detail information was shown in Table 4.

**Table 4:** Comparison of Endothelial Function between the Two Groups (x̄±s, n=46). *Compared to before treatment, P<0.05; #compared to the control group, P<0.05.

Group	ET-1 (pg/mL)		sICAM-1 (ng/mL)		VEGF (ng/L)	
	Before treatment	After treatment	Before treatment	After treatment	Before treatment	After treatment
Observation<br>group	75.26±4.12	51.84±3.08^*#^	304.86±26.91	127.55±21.03^*#^	123.51±23.81	203.66±31.87^*#^
Control group	75.38±4.20	59.75±3.54^*#^	305.11±25.72	156.97±23.44^*^	124.17±22.53	184.54±25.16^*^
*t*	0.138	11.433	0.046	6.336	0.137	3.194
P-value	0.890	<0.001	0.964	<0.001	0.892	0.002

### Comparison of immune function cells between the two groups

**Figure 1:**
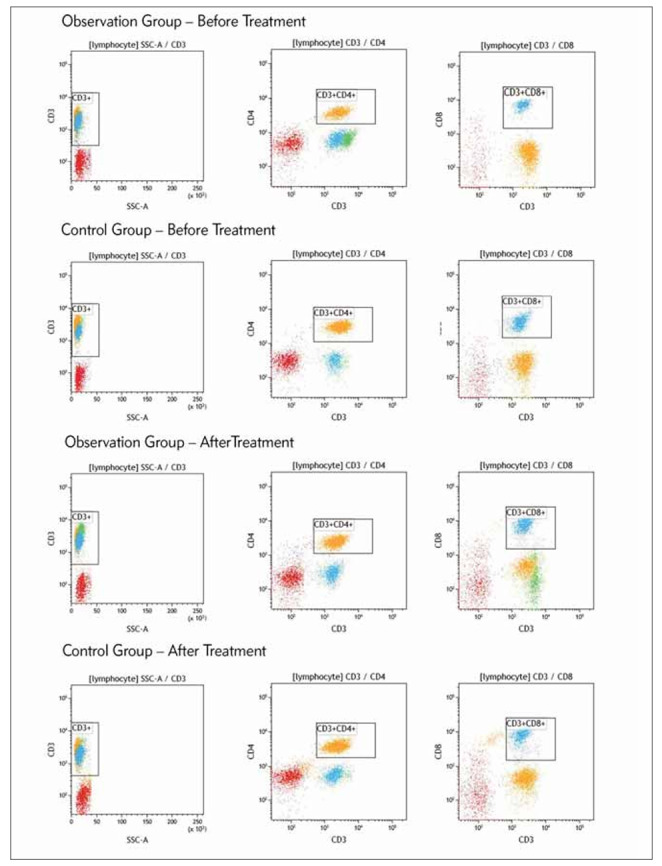
Flow Cytometry Analysis of Changes in Immune Cell Subsets in Peripheral Blood of Patients in Two Groups Before and After Treatment: Left: CD3+ Lymphocyte Subpopulation; Middle: CD3+CD4+ Lymphocyte Subpopulation; Right: CD3+CD8+ Lymphocyte Subpopulation.

Using flow cytometry, changes in immune cell function were detected in both groups before and after treatment. Before treatment, there were no significant differences in the proportions of CD3+ lymphocytes, CD3+CD4+ lymphocytes, CD3+CD8+ lymphocytes, and the immune function index (CD3+CD4+/CD3+CD8+ ratio) in peripheral blood between the two groups (P > 0.05). However, after treatment, the proportions of CD3+ lymphocytes, CD3+CD4+ lymphocytes, and the CD3+CD4+/CD3+CD8+ ratio increased significantly in both groups (P < 0.05). Moreover, the observation group showed significantly higher proportions of CD3+ lymphocytes, CD3+CD4+ lymphocytes, and a higher CD3+CD4+/CD3+CD8+ ratio compared to the control group (P < 0.05). The detailed information was presented in Figure 1 and Table 5.

**Table 5:** Comparison of Immune Function Cells between the Two Groups (x̄±s, n=46). *Compared to before treatment, P<0.05; ^#^compared to the control group, P<0.05.

Group	CD3+ (%)		CD3+<br>CD4+ (%)		CD3+<br>CD8+ (%)		CD3+CD4+/<br>CD3+CD8+ (%)	
	Before<br>treatment	After<br>treatment	Before<br>treatment	After<br>treatment	Before<br>treatment	After<br>treatment	Before<br>treatment	After<br>treatment
Observation<br>group	56.76±3.55	72.35±5.07^*#^	26.42±3.67	41.14±3.33^*#^	25.56±2.98	27.98±3.07	0.95±0.11	1.42±0.18^*#^
Control<br>group	56.34±3.87	65.23±5.26^*^	25.93±3.11	32.45±3.48^*^	26.67±2.58	28.86±3.14	0.93±0.12	1.15±0.19^*^
*t*	0.324	15.636	0.242	12.747	0.119	0.535	0.106	10.116
P-value	0.425	<0.001	0.364	<0.001	0.385	0.414	0.289	<0.001

### Comparison of adverse reactions between the two groups

There were no significant differences in the occurrence rates of adverse reactions between the two groups (P > 0.05). The detailed information was shown in Table 6.

**Table 6:** Comparison of Adverse Reactions between the Two Groups (n (%), n=46).

Group	Nausea	Vomiting	Headache	Gastrointestinal<br>bleeding	Digestive tract<br>bleeding	Diarrhea	Total incidence of<br>adverse reactions
Observation group	2	3	1	2	1	3	12 (26.09)
Control group	1	2	2	1	2	1	9 (19.56)
χ^2^							0.555
P value							0.456

### Comparison of the incidence of major cardiovascular events between the two groups

In the observation group, there was only 1 case of myocardial infarction, resulting in a major cardiovascular event incidence rate of 2.17%. In the control group, there were 3 cases of stroke, 2 cases of myocardial infarction, and 1 case of cardiovascular death, resulting in a major cardiovascular event incidence rate of 13.04%. Upon comparison, the major cardiovascular event incidence rate in the observation group was lower than that in the control group (χ^2^=3.866, P=0.049).

## Discussion

Research has shown that high uric acid levels are the end product of purine metabolism and the underlying cause of acute gout attacks, which is related to CHD [11]. In recent years, the rapid aging of the population and improved living standards in China have led to an increasing incidence of CHD. This has resulted in a substantial rise in the number of patients with acute gout attacks complicating CHD. This not only poses a significant threat to the lives of patients but also increases the economic and societal burdens. Therefore, adopting safe and effective treatment measures is of great importance for controlling disease progression and improving prognosis. There is a growing interest in anti-inflammatory treatment for CHD patients with acute gout attacks, as recent studies have highlighted the role of inflammation in the development of atherosclerosis [12]. Colchicine, as a cost-effective anti-inflammatory drug, has a wide range of anti-inflammatory effects. Hence, this study applied colchicine as an adjunctive therapy for CHD patients with acute gout attacks, aiming to explore its effects on disease control and its impact on relevant inflammatory factors and vascular endothelial function factors.

In the current study, the observation group showed a higher overall clinical effective rate compared to the control group, indicating that colchicine adjuvant therapy is beneficial for improving the clinical efficacy in patients with CHD and acute gout attacks. It plays a positive role in controlling the further development of the disease. This effect may be related to the anti-inflammatory, immunosuppressive, anti-fibrotic, and cardiovascular protective properties of colchicine. Additionally, a study conducted by Wang Pengfei et al. [13] also demonstrated that colchicine had good efficacy in treating patients with angina pectoris and acute gout, leading to improvements in gout and serum markers. This finding aligns with the results of the current study.

CHD is a chronic vascular inflammatory condition characterized by the accumulation of subendothelial lipoproteins, triggering abnormal immune responses and resulting in the formation of inflammatory plaques [14]. Furthermore, research has indicated a close association between inflammatory res ponses and both CHD and acute gout attacks [15] [16]. Serum NALP3 plays a critical role in the inflammatory response, as it activates caspase-1, which in turn regulates the maturation of interleukins (IL)-1β and IL-18 in white blood cells. This activation leads to the release of other pro-inflammatory cytokines, exacerbating inflammation and contributing to the development of CHD and acute gout attacks. In atherosclerosis, infiltrating inflammatory cells release substantial amounts of MMP-9, enhancing the activity of other inflammatory mediators and further amplifying the inflammatory response, ultimately resulting in abnormally elevated levels of MMP-9 in patients [17]. Additionally, MMP-9 can specifically bind to extracellular matrix components, promoting the formation of unstable plaques [18]. MMP-13, another collagenase, is involved in various inflammatory reactions and can severely impair heart function by degrading native fibrillar collagen, leading to cardiac atrophy [19]. In this study, after treatment, the observation group exhibited reduced levels of NALP3, MMP-9, and MMP-13 compared to the control group, suggesting that adjunctive treatment with colchicine is beneficial for alleviating inflammation and lowering serum NALP3, MMP-9, and MMP-13 levels in patients with CHD and concurrent acute gout attacks. It is likely that colchicine aggregates within neutrophils, interfering with their adhesion, recruitment, and deformation by affecting relevant chemotactic factors, thereby reducing urate crystal-induced inflammatory responses. Furthermore, colchicine can inhibit pore formation induced by urate receptor activation, reducing inflammasome activation [20]. Moreover, colchicine can stimulate M2 macrophages to increase the expression of transforming growth factor-beta (TGF-β), which limits the proliferation and activity of smooth muscle cells and fibroblasts, thereby promoting the resolution and healing of plaque inflammation [21]. This leads to a reduction in inflammatory responses and a decrease in serum NALP3, MMP-9, and MMP-13 levels.

Vascular endothelial dysfunction plays a role in the development of CHD combined with acute gout attacks. ET-1, as a vasoconstrictive peptide, can induce vascular constriction. Elevated levels of ET-1 can led to insufficient myocardial blood supply. sICAM-1 can increase plaque vulnerability and is often used as an important biochemical indicator to assess the severity of CHD [22]. VEGF promotes endothelial cell regeneration and angiogenesis. When endothelial function is impaired, it can lead to coronary artery narrowing and myocardial ischemia and hypoxia, resulting in reduced VEGF levels. This study demonstrates that after treatment, the observation group had lower levels of ET-1 and sICAM-1 compared to the control group, while VEGF levels were higher than in the control group. This suggests that adjunctive treatment with colchicine is beneficial for improving endothelial dysfunction in patients with CHD and concurrent acute gout attacks. This may be due to colchicine’s effective uric acid-lowering properties, which reduce urate crystal deposition, thereby minimizing damage to the vascular endothelium and preventing thrombus formation. Furthermore, colchicine can inhibit the interaction between white blood cells and platelets, reduce endothelial cell selectin expression, and exert anti-platelet aggregation effects, thus protecting endothelial function [23]. In this study, after treatment, the observation group had lower levels of LVPWT and LVEDD while LVEF was higher compared to the control group, indicating that adjunctive colchicine treatment in patients with CHD and concurrent acute gout attacks is associated with improved heart function. This improvement may be attributed to colchicine’s anti-inflammatory properties and its role in improving endothelial function. The study also found that the observation group had a lower rate of major cardiovascular events compared to the control group, which is consistent with previous research [20]. This suggests that adjunctive colchicine therapy has a positive impact on reducing the occurrence of major cardiovascular events. Additionally, research by Fiolet et al. [24] also demonstrated that colchicine treatment can reduce the risk of cardiovascular events in stable CHD patients.

Furthermore, the study showed that the immune cell subsets (CD3+ lymphocytes, CD3+CD4+ lymphocytes ratio) and immune index (CD3+CD4+/CD3+CD8+ ratio) in the observation group were significantly higher than in the control group, indicating that colchicine adjunctive therapy can enhance the immune response recovery in patients with CHD and concurrent acute gout attacks. Finally, the study found no significant difference in the incidence of adverse reactions between the two groups, reaffirming the safety of adjunctive colchicine therapy. However this paper still has some shortcomings. Firstly, the adequacy of the sample size was not calculated for this study, however we collected a more than adequate number of patients. Secondly this is a single centre study, which may limit the application of the results of this study. In the future we will conduct a multi-centre study.

In conclusion, adjunctive treatment with colchicine in patients with CHD and concurrent acute gout attacks has shown superior therapeutic efficacy. It helps reduce inflammation, improve endothelial function and cardiac function, promotes immune recovery, and reduces the occurrence of major cardiovascular events.

## Dodatak

### Funding

This study was funded by the Medical Science Research Project of Hebei Province (20232111).

### Conflict of interest statement

All the authors declare that they have no conflict of interest in this work.

### Contribution

Both authors contributed equally to the article.
